# UV Response of the Green Fluorescent Protein Chromophore: Insights from Ab Initio Nonadiabatic Simulations

**DOI:** 10.1021/acs.jpclett.6c00415

**Published:** 2026-04-21

**Authors:** Wutthinan Thongyod, Tingyao Lu, Yuanming Bai, Chutintorn Punwong, William J. Glover

**Affiliations:** † NYU-ECNU Center for Computational Chemistry at NYU Shanghai, 3663 Zhongshan Road North, Shanghai 200062, China; ‡ School of Science, and Research Center for Theoretical Simulation and Applied Research in Bioscience and Sensing, 65133 Walailak University, Nakhon Si Thammarat 80160, Thailand; # Shanghai Frontiers Science Center of Artificial Intelligence and Deep Learning, 447103 NYU Shanghai, 567 West Yangsi Road, Shanghai 200127, China; § Department of Chemistry, New York University, New York, New York 10003, United States; ∥ Division of Physical Science, Faculty of Science, 26686 Prince of Songkla University, Hat Yai, Songkhla 90112, Thailand; ⊥ Center of Excellence for Trace Analysis and Biosensor, 26686 Prince of Songkla University, Songkhla 90112, Thailand

## Abstract

The green fluorescent protein (GFP) is widely used in imaging organisms at the subcellular level. However, upon exposure to UV or intense visible light, GFP undergoes irreversible reactions, altering its photocycle, which are believed to precede via photooxidation of the chromophore. The mechanism of this process is not well understood, even in the gas phase, with competing interpretations of photoelectron experiments on the isolated chromophore arguing either for nonadiabatic decay or intramolecular vibrational energy redistribution (IVR) and autoionization from an initially populated S_3_ shape resonance. To address the controversy, we simulated the excited-state dynamics and time-resolved photoelectron spectroscopy (TRPES) of the GFP chromophore with ab initio multiple spawning and an on-the-fly multiconfigurational electronic structure using our dynamically weighted complete active space self-consistent field method. Our simulations show excellent agreement with experimental TRPES and reveal that S_3_-S_2_ nonadiabatic transitions do occur on an ultrafast time scale that can compete with autoionization; however, the conversion between the shape and Feshbach states occurs primarily adiabatically on the S_2_ state. Furthermore, the threshold energies of the shape and Feshbach resonances are very similar, with a low barrier separating these regions of the PES, leading to both states being populated and reversibly interconverting within 50 fs of the initial photoexcitation. As a result, rapid autoionization still precedes via the shape resonance. The picture that emerges thus reconciles the two competing views of the GFP chromophore’s UV response: both internal conversion and IVR from the shape resonance state are operative. Our findings of the involvement of the Feshbach state suggest new strategies to engineer FP chromophores with tailored photostabilities.

## Graphical abstract

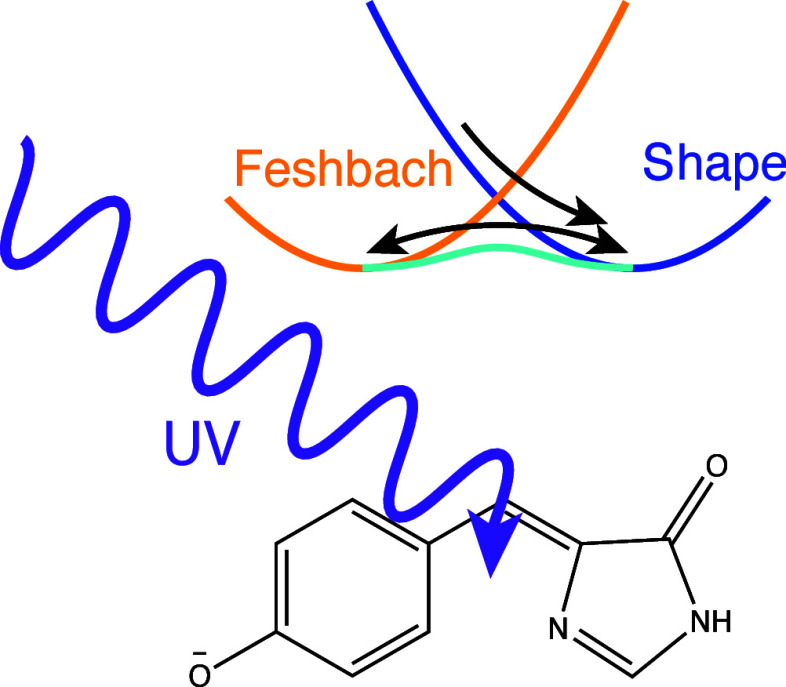

Green fluorescent protein (GFP) was first extracted from the jellyfish *Aequorea victoria* in 1962 and has revolutionized many fields of biology due its use in imaging organisms at the subcellular level.^1^ Our understanding of GFP (and its variants) photophysics following excitation with visible light is now quite mature.^2–4^ Nevertheless, one issue that limits the use of GFP is photostability: upon excitation with UV or intense visible light, irreversible photoredox reactions occur, such as oxidative decarboxylation, which modify the absorption and emission spectrum.^5–8^ On the one hand, this can complicate a quantitative analysis of GFP’s emission when used in two-photon excitation spectroscopy.^9,10^ On the other hand, it allows for FPs to be used as light-induced electron donors for monitoring or adjusting redox processes in cells.^11^

A mechanistic understanding of GFP’s photo-oxidation will provide design rules for new FPs with improved photostability or tailored photoredox potentials, yet basic details remain unclear. In particular, an open question is the nature of the electronic state(s) that initiate(s) photo-oxidation. Since GFP is photostable under low-intensity visible light conditions, but undergoes phototransformation under UV irradiation,^6^ it is understood that photodamage occurs from a high electronic state (above the bright S_1_ ππ* state responsible for fluorescence). Furthermore, it has been demonstrated that a two-photon absorption of visible light leads to a similar photoconversion of DsRed with the chromophore in its anionic form,^12^ which indicates that the chromophore anion is directly involved in the reaction.

Given the complications of overlapping UV absorption bands from the protein environment, a bottom-up approach to understanding GFP’s photo-oxidation has been popular, focusing on the photoresponse of the isolated *p*-hydroxybenzilidene-2,3-dimethylimidazolinone (HBDI) chromophore anion and related biomimetic molecules.^13–17^

The key electronic states of the HBI anion (the demethylated analog of HBDI) are depicted in Figure 1. In particular, there is a vertical S_3_ state corresponding to a shape resonance at ∼320 nm (∼3.9 eV), which lies about 0.2 eV above a S_2_ Feshbach resonance,^15^ and one or both of these states are believed to be involved in the photo-oxidation. Lower lying dark nπ* states, not shown here, have been predicted to exist,^15^ but they are not believed to be involved in the photo-oxidation of GFP.

**Figure 1:**
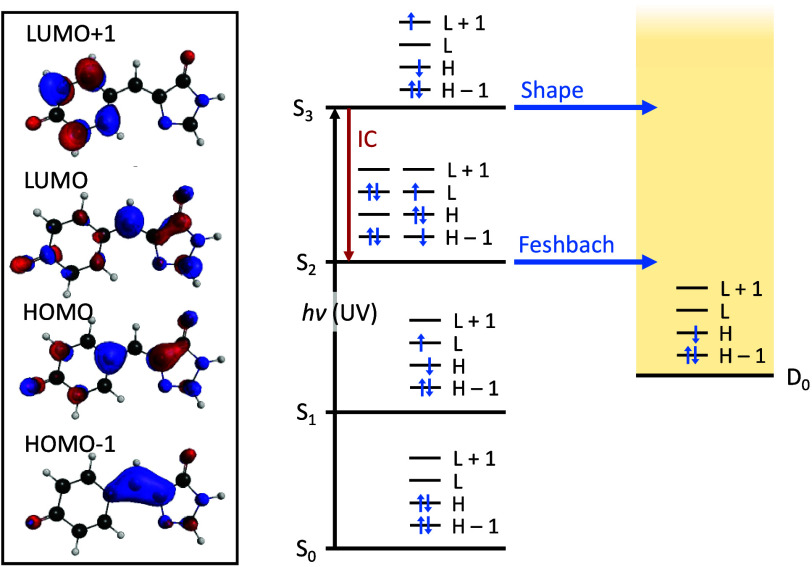
Dominant π orbitals, electron configurations, and energy levels proposed to be involved in the initial UV photoresponse of HBI. Arrows indicate possible pathways following an optically allowed S_3_ ← S_0_ UV excitation.

The shape-resonance nature of the S_3_ state prompted Bochenkova et al. to propose a GFP phototransformation mechanism in which the anionic chromophore is initially photo-oxidized, which then initiates an irreversible oxidative Kolbe decarboxylation of the nearby Glu222 residue.^5,6,15^ Within this proposal, in the gas phase, the initial photo-oxidation occurs via autodetachment, while in the protein environment, it occurs via a charge-transfer reaction, possibly forming a hydrated electron.^15^

The proposal that photo-oxidation initiates from S_3_ inherently assumes that autoionization (in the gas phase) or charge transfer (in the protein) occurs on a time scale faster than internal conversion (IC) of S_3_, despite the S_2_ state being energetically close. This assumption was called into question by West et al, who used two-dimensional and time-resolved photoelectron spectroscopy (TRPES) of the gas-phase HBDI anion to assign a photoelectron signal from S_2_ and an S_3_ to S_2_ IC time scale of <40 fs.^16^ In particular, using a 4.1 eV pump and 1.55 eV probe, they observed additional photoelectrons between 1.5 and 3.0 eV of electron kinetic energy (eKE) at positive pump–probe time delays compared to negative time delays (see Figure 2 of ref 16 and Figure 2(a) below). The decay of photoelectron intensity with increasing pump–probe time delays was not uniform across the eKE range, with a high-eKE window between 2.5 and 3.0 eV (gray-shaded area) decaying on *a* < 40 fs time scale (within their time resolution), and a lower eKE window between 1.5 and 2.5 eV (blue-shaded area) decaying on a ∼55 fs time scale. The faster decay of the high-eKE window was assigned to IC between the S_3_ and S_2_ states, suggesting that the predominant doorway state of GFP’s photo-oxidation is not the shape state, but instead the Feshbach state.

**Figure 2:**
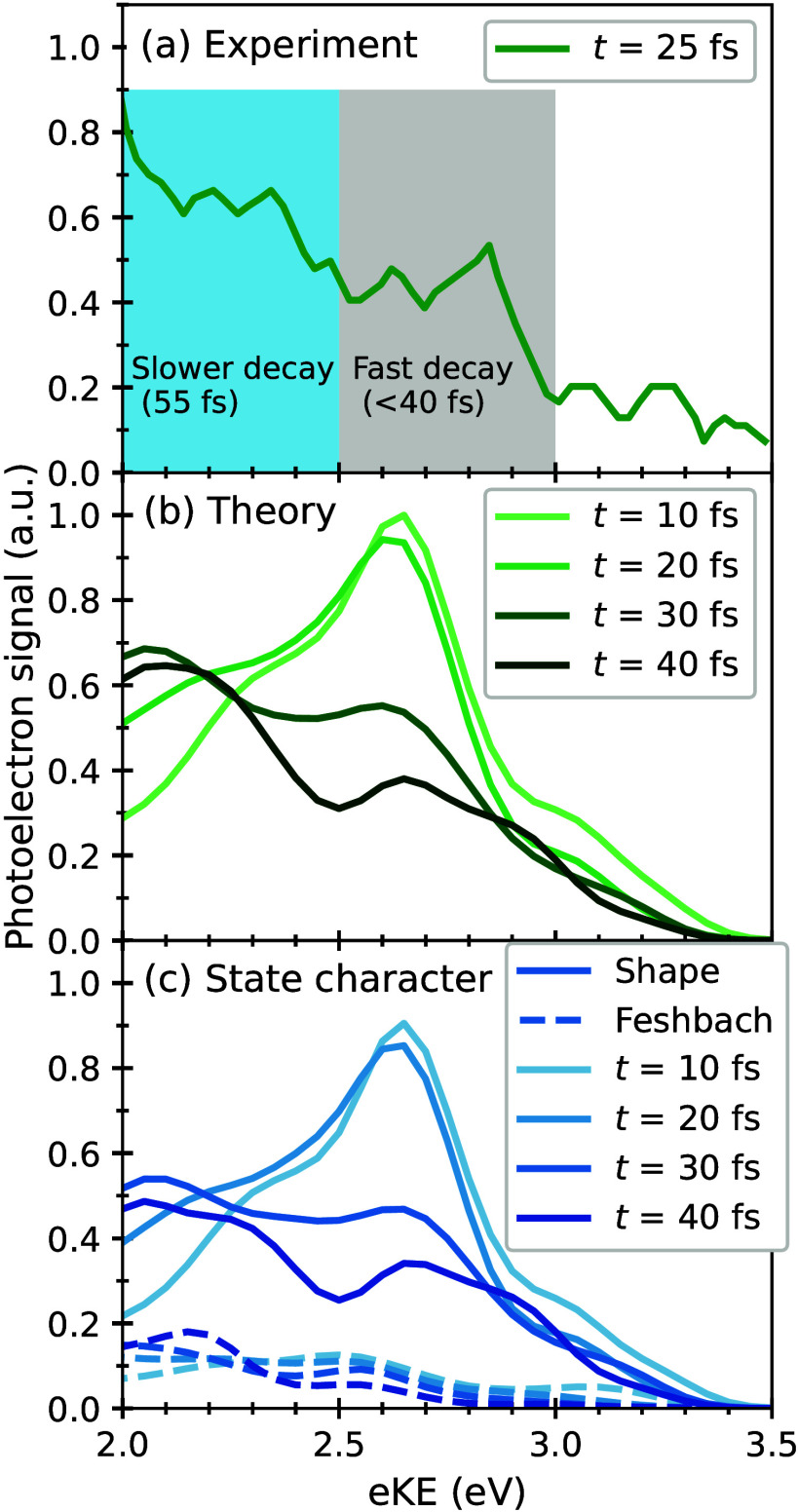
TRPES of the gas-phase HBI anion with pump and probe photon energies of 4.1 and 1.55 eV respectively. (a) Experimental data, digitized from Figure 2­(a) of ref 16. To aid the comparison to the theoretical spectra, the electron kinetic energy range is restricted to 2.0 to 3.5 eV. (b) Theoretical spectra from the DW­(4,4 eV)-CASSCF­(4,5)/6-31G* simulations described in this paper. (c) Breakdown of the TRPES in terms of state character.

However, the interpretation of HBDI’s photoelectron spectroscopy is controversial: Bochenkova assigned the decay of the high eKE feature in the TRPES of HBDI not to S_3_/S_2_ IC, but instead to intramolecular vibrational energy redistribution (IVR) and autodetachment from the shape state.^17^ However, the possibility that IC, IVR, and autodetachment occur on similar time scales cannot be ruled out and motivates the current work. In particular, we seek to address the roles of IVR and IC in the UV response of the GFP chromophore and to what extent they are revealed by TRPES.

Previously we showed that there exists an energetically accessible conical intersection (CI) between S_3_ and S_2_ of the gas-phase GFP chromophore anion with a geometry only slightly distorted from the Franck–Condon structure.^18^ This supports the likelihood of ultrafast IC between the states; however, nonadiabatic dynamics simulations are needed to determinate the time scale and outcome of this process. Thus, in this work, we use Ab Initio Multiple Spawning (AIMS) with a dynamically weighted complete active space self-consistent field (DW-CASSCF) multiconfigurational electronic structure to explore the excited-state dynamics of the GFP chromophore anion following UV excitation.^19–21^ To make a connection with experiment, we compute TRPES observables from the simulations. Details of our methodology are provided in the Computational Methods section.

In what follows, we consider the simpler HBI analogue of the GFP chromophore, but we showed previously that the energy levels are essentially unchanged relative to HBDI.^22^ Furthermore, as we will see below, the dominant UV photoresponse involves motions in the phenoxide ring and allyl bridge, which are preserved between the molecules, and therefore, we expect our dynamical predictions to extend to HBDI.

The adiabatic population dynamics following an excitation to S_3_ are shown in Figure 3(a), where we clearly see S_3_-S_2_ nonadiabatic transitions on a ∼20 fs time scale. This ultrafast time scale is consistent with the presence of a S_3_-S_2_ CI of peaked character in the vicinity of the Franck–Condon region that we previously discovered,^18^ and which we replot in Figure 5 below on an expanded scale. Although included in our electronic structure calculations, no transitions to S_4_ or higher were noted. We do observe a small population on S_1_ by 50 fs, suggestive of an energetically accessible S_2_-S_1_ CI; however, we leave the exploration of this to future work.

**Figure 3:**
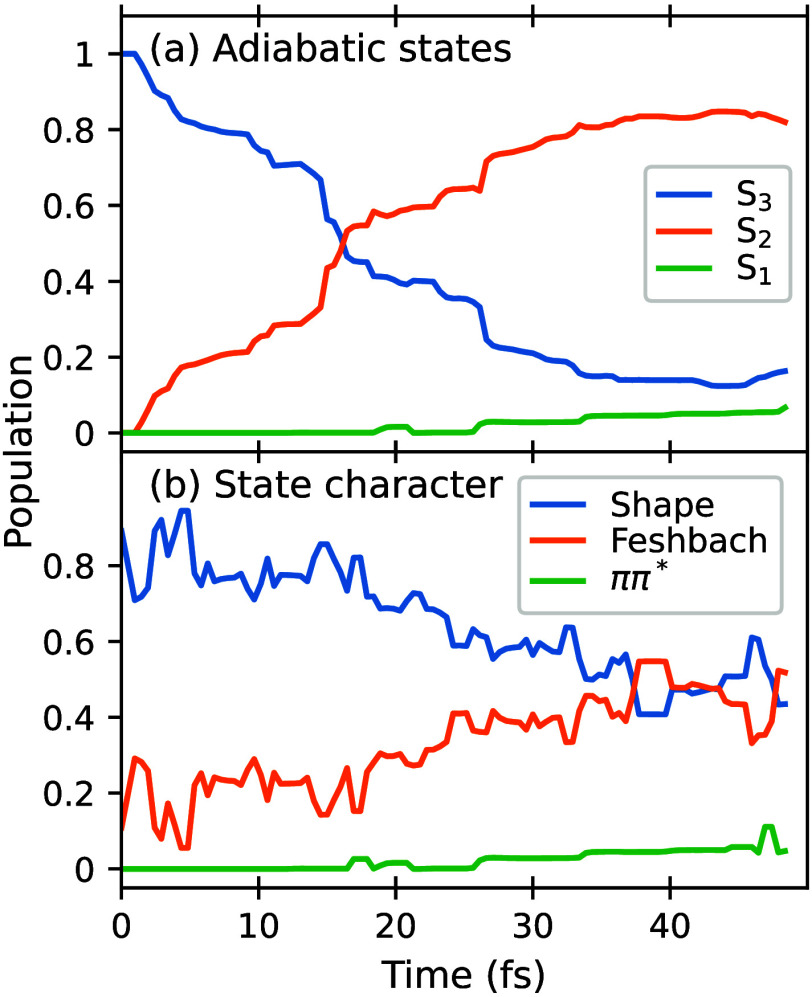
(a) Normalized adiabatic population dynamics of HBI following excitation. (b) Population dynamics based on the character of the state (see text for definition). Note: the 20% Feshbach population at *t* = 0 fs arises from initial conditions in which the shape and Feshbach state ordering is reversed and in which the Feshbach state acquires some oscillator strength from mixing with the Shape resonance.

The adiabatic population dynamics alone does not report on the shape resonance’s lifetime since its character could be preserved through the change of the adiabatic state from S_3_ to S_2_. To monitor the character of the populated excited state, we consider a simple classification based on the ground-to-excited oscillator strength and Dyson norm for ionization from any of the lowest three singlet excited states to the D_0_ state, summarized in Table 1 below.

**Table 1:** Oscillator Strengths and Dyson Norms for the Lowest Excited States of HBI

transition	Osc. str. (au)	Dyson norm	identifier
Bright ππ*	1.540	0.721	Largest Osc. str.
Feshbach	0.0874	0.345	Not largest Osc. str. and smallest Dyson norm
Shape	0.195	0.607	Not largest Osc. str. and not smallest Dyson norm

After relabeling the states at each time step of each trajectory according to the classification of Table 1, we find the character-based population dynamics shown in Figure 3(b). The shape-resonance character is seen to persist noticeably longer than the lifetime of the S_3_ state; nevertheless, there is a clear conversion to the Feshbach state, reaching a roughly equal mixture of these states at the end of our simulation.

At first glance, our results appear to support the conclusions of West et al. that the ultrafast decay of the initially populated shape state is due to IC;^16^ however, our analysis reveals that the lifetime of the shape state is clearly longer than the <40 fs time scale for the decay of the high-KE TRPES region they assigned to this state. To understand the origin of this discrepancy, we simulated the TRPES from our trajectories, the results of which are shown in Figure 2(b).

The simulated TRPES shows that the photoelectron signal between 2.5 and 3.0 eV is seen to decay on a time scale faster than the photoelectron signal between 2.0 and 2.5 eV in line with the experimental observations.^16^ Breaking down the spectrum into contributions from shape vs Feshbach character, shown in panel (c), we see that the TRPES spectral dynamics are essentially entirely probing the shape state—the Feshbach state is dark to one-photon ionization to D_0_, as expected from its low Dyson norm, and ionization to D_1_ is not observed in this eKE range. As a result, the decay of the TRPES spectrum in the 2.5–3.0 eV window is not solely due to IC, but arises mainly from the shape spectrum broadening and its initial peak at 2.6 eV being replaced by a peak at 2.1 eV (within this energy window) due to IVR on a <30 fs time scale.

To better understand the molecular states probed in the TRPES spectrum, we performed critical point optimizations at the same DW-CASSCF electronic structure theory level used in the dynamics, as summarized in Figure 4(a). While the shape state is the S_3_ adiabatic state in the Franck–Condon (FC) region, there is an energetically accessible S_3_-S_2_ minimal energy conical intersection (MECI) with a planar geometry not much distorted from the FC geometry. As a result, HBI undergoes rapid conversion from S_3_ to S_2_, but Figure 3 reveals that this occurs largely *diabatically*, preserving the initial shape character. We identify a minimum on the S_2_ surface with shape character (S_2_ Shape Min.), leading to a rather large reorganization energy of 0.66 eV (the difference between the FC vertical shape energy and shape S_2_ minimum energy) from the FC region and an even larger change of the shape state energy relative to the vertical D_0_ state (1.53 to 0.75 eV). These results, along with the qualitative energetic ordering of the other critical points, hold up to higher-level XMS-PT2/cc-pVTZ electronic structure calculations shown in panel (b). Stabilization of the shape state relative to the D_0_ state due to IVR leads to a rapid reduction of the eKE in the TRPES spectrum over the first 30 fs. As a result, the initial changes in the TRPES spectrum can be largely assigned to IVR of the shape state rather than IC, supporting Bochenkova et al.^17^

**Figure 4:**
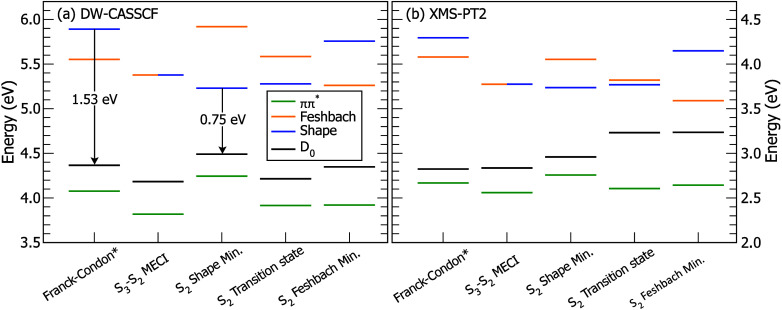
Electronic energies at relevant critical points on the potential energy surfaces of HBI. Panel (a) shows results at the DW­(4,4 eV)-CASSCF­(4,5)/6-31G* level of theory, while panel (b) shows results at the SA-4-CAS­(4,5)­XMS-PT2/cc-pVTZ level. All energies are relative to S_0_ at the optimized ground-state structure at the respective level of theory. Arrows indicate the difference in energy between the shape state and ionized D_0_ state. *The Franck–Condon structure is displaced by 0.05 Å along the S_0_-S_3_ gradient difference vector so as to place the shape state above the Feshbach state and to mimic photoexcitation to the blue of the shape state’s action spectrum peak.

However, IVR is not the whole story. The character-based population curves in Figure 3 reveal that IC *does* occur between shape and Feshbach, but this happens primarily adiabatically on the S_2_ state, rather than during transit through the S_3_-S_2_ CI. Furthermore, the conversion appears to be reversible (indicated by anticorrelated oscillations in the populations of shape and Feshbach states), resulting in a mixture of the states by the end of our simulations. To understand this, we explore the S_3_-S_2_ PES in the vicinity of the CI by extending the branching plane to larger displacements than considered in ref 18. The result is shown in Figure 5, where we see that the peaked intersection resides at the center of a Mexican hat potential, characteristic of a Jahn–Teller intersection.

**Figure 5:**
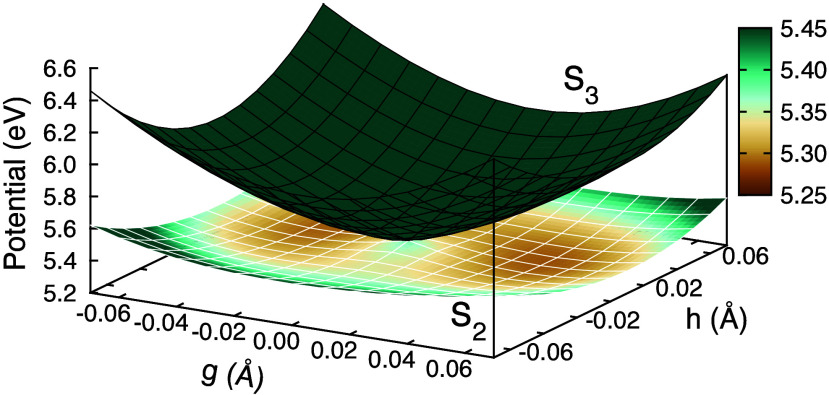
Potential energy surfaces in the branching plane of the S_3_/S_2_ minimal energy conical intersection computed with DW­(4,4 eV)-CASSCF­(4,5)/6-31G*.

The adiabatic conversion of the shape and Feshbach states can thus be understood as wave packet dynamics spreading out in the bottom ring of the Mexican-hat potential. The low barriers and near degeneracy of the ring explain the ultrafast conversion time scale and reversible nature of the internal conversion. This picture is supported by the energies of fully optimized critical points, shown as the solid lines in the energy-level diagram of Figure 4(a), where two minima are seen on S_2_ with shape and Feshbach character. The energies of these minima essentially match those of Figure 5, indicating that nuclear relaxation is indeed predominantly within the g-h branching plane (gradient difference and nonadiabatic coupling vectors, respectively) of the S_3_/S_2_ intersection. As determined previously, these displacements primarily involve the Kekule and breathing modes of the phenoxide ring and allyl bridge stretching motions.^18^

The picture that emerges from our simulations, summarized in Figure 6, thus reconciles and refines the competing interpretations of the anionic GFP chromophore’s photoelectron spectroscopy. In agreement with West et al.’s interpretation,^16^ a conversion between the shape and Feshbach states occurs on an ultrafast time scale that competes with autoionization; however, this proceeds adiabatically on the S_2_ state and leads to an electronic population roughly equally split between shape and Feshbach character. Then, given the slower time scales for autoionization from Feshbach states,^17^ autoionization is expected to occur from the shape state, in agreement with the interpretations of Bochenkova et al.^15,17^ Given the rapid interconversion of shape and Feshbach populations, autoionization from the shape also depletes the Feshbach state.

**Figure 6:**
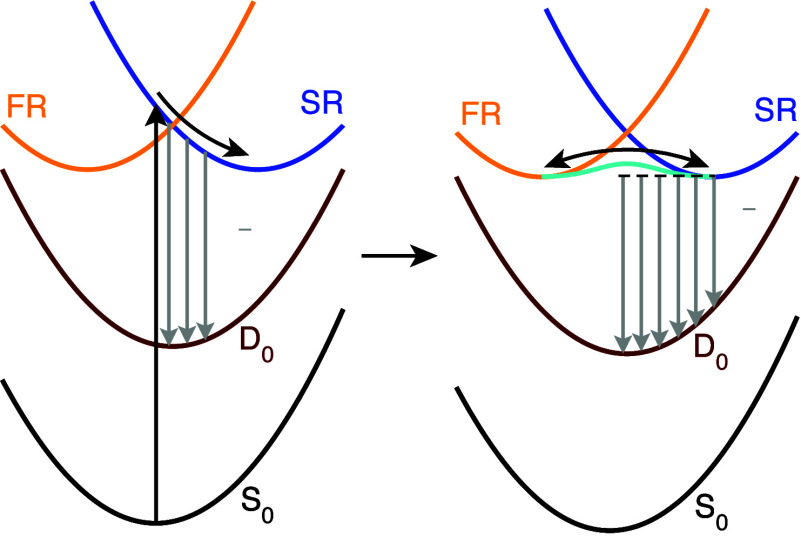
Schematic of the UV photoresponse of the GFP chromophore anion. Left: After excitation to the S_3_ shape resonance (SR), a direct electron detachment channel arises from the initially populated state to D_0_ (gray arrows). In competition with direct detachment, excited-state dynamics leads the molecule to pass through a conical intersection, while largely retains its shape character. Right: within 50 fs following photoexcitation, excited-state dynamics on the S_2_ adiabat leads to conversion between the shape and Feshbach resonance (FR) states. Delayed electron detachment via autoionization from the shape state then depletes the excited singlet population.

Our finding that the shape and Feshbach states undergo rapid interconversion suggests that the Feshbach state could serve as a doorway state to protection from rapid photo-oxidation. While this is not the case in the isolated GFP anion since the Feshbach and shape states have similar threshold energies and rapidly interconvert, one can envisage modulating their relative energies by tuning the environment or via chemical modifications of the chromophore. This will allow for FPs with tailored photo-oxidation rates or photoredox potentials. Work in this direction is in progress.

Following a bottom-up approach, we chose to focus on the UV-initiated excited-state dynamics of the gas-phase GFP chromophore that precedes photooxidation. It is natural to anticipate how our findings might relate to the dynamics of the chromophore in the protein. Since we find that IVR and IC occur within 50 fs, significant protein reorganization will not take place on this time scale. Furthermore, our critical point calculations reveal that the chromophore in its S_2_ and S_3_ states stays largely planar; therefore, steric effects of the protein are also likely not important. We thus expect that studies of the UV-initiated intrinsic early time dynamics of the isolated chromophore can be useful also for understanding the photophysics of the protein.

Nevertheless, the protein electrostatic and hydrogen-bonding environment could alter the vertical and adiabatic energies of the states that correlate with the gas-phase shape and Feshbach states, which could modify the mechanism of photodeactivation revealed by gas-phase studies. Bochenkova et al. found that the shape state’s vertical excitation shifted from 325 nm (3.8 eV) in the gas phase to 280 nm (4.4 eV) in the protein.^15^ The gas-to-protein shift of the Feshbach state was not reported, but given the greater charge-transfer character of the shape state, the shift for the Feshbach state is expected to be smaller in magnitude. This will lead to an increase in the shape state’s energy relative to the Feshbach state, which, based on our picture, would lead to more complete IC from shape to Feshbach. We are thus currently testing this hypothesis by conducting excited-state dynamics simulations of the chromophore in its protein environment, and we will report those results in a future publication.

## Computational Methods

We performed excited-state dynamics simulations of the deprotonated HBI anion using the MPI interface between development versions of TeraChem^23^ and the ab initio multiple spawning package.^19,24^ The electronic structure was solved on-the-fly using our dynamic weighting scheme for the complete active space self-consistent field (DW-CASSCF) method.^18,25^ We showed previously that DW-CASSCF improves the stability of nonadiabatic excited-state dynamics simulations over regular state-averaged CASSCF.^25^ The active space consisted of 4 electrons in 5 orbitals using the 6-31G* basis. Following our previous studies,^18,22^ 4 states were state averaged with equal weights, while higher-lying states were included in the state averaging with weights that decayed following a cubic-spline function of their energy gap to S_3_. The width of the cubic spline was 4 eV. In total, 12 excited states were included in the simulation.

Formally, the shapes and Feshbach states under consideration are electronic resonances above the ionization threshold. We did not attempt to describe the electronic continuum or the finite lifetime of the electronic states due to autoionization, instead relying on the compact nature of the 6-31G* basis to capture the main features of the electronic wave functions in the immediate vicinity of the molecule. We confirmed that although the ground-to-excited state energies of HBI are significantly overestimated at the CASSCF level, due largely to a neglect of dynamic electron correlation, the relative excited-state energies at key critical points are in good agreement with high-level multireference perturbation theory calculations at the SA-4-CAS­(4,5)­XMS-PT2/cc-pVTZ level (and SA4-CAS­(3,5)-XMS-PT2 for the ionized neutral states) using the BAGEL quantum chemistry package^26^ (see Figure 4). We also verified that augmentation of the basis set with diffuse functions, while lowering the excitation energies of all states by around 0.33 eV, does so uniformly across all relevant critical point geometries (see ). Taken together, this suggests a good degree of parallelity between excited-state potential energy surfaces computed with DW-CASSCF without diffuse functions and high-level XMS-PT2 with diffuse functions. Thus, an improvement of the electronic structure method is not expected to qualitatively change the conclusions of our study.

To simulate time-resolved photoelectron spectra, we followed the protocol of ref 27, while mimicking the experimental conditions of a 4.1 eV pump and 1.55 eV probe.^16^ The initial ground-state wavepacket was expanded in a basis of 20 Gaussian trajectory basis functions (TBF), each sampled from the Wigner distribution of HBI under the harmonic approximation using frequencies calculated at the MP2/6-31G** level. The initial TBFs were further enforced to have a ground-excited vertical energy gap of 0.216 ± 0.0005 hartree (5.88 ± 0.014 eV) in order to mimic the experimental pump pulse that was slightly blue of the Franck–Condon (FC) S_3_ action-spectra peak,^16^ taking into account that the anionic CASSCF excitation energies are overestimated relative to the neutral D_0_ state. The wavepacket was then projected on to the excited states, with each TBF amplitude weighted according to its ground-excited oscillator strength, and then uncoupled, following the independent first-generation approximation.^24^ Due to the pump energy window, only the S_3_ state was populated among the initial TBFs. Ab Initio multiple spawning dynamics^19,24^ was then propagated with adaptive timesteps of 20 au by default, but steps were rejected and repeated with half the previous time step if CI vector overlaps involving the occupied state between steps were not diagonally dominant or energy discontinuities greater than 0.006 hartree (0.16 eV) were detected.

To generate TRPES spectra, the anionic wavepacket was projected to the neutral doublet states. The electron kinetic energy for each TBF’s contribution to the TRPES was evaluated as

1 $$EKE=h\nu_{probe}-\left(E_{neut}-E_{anion}+\Delta \right)$$

where *hν* = 1.55 eV and Δ is an energetic shift of +2.0 eV applied to the neutral doublet state to bring the TRPES spectrum into alignment with experiment. This shift, also applied in Figure 4(a), mainly corrects for the overestimation of the anionic excited-state energies at the CASSCF level (and also from the lack of diffuse functions in our calculations). The ionized neutral states came from CASCI calculations, with orbitals frozen to the anionic DW-CASSCF electronic structure. Dyson norms between the anionic and neutral states, needed for the TRPES intensities, were computed in BAGEL.^26^ Finally, the raw TRPES signals from the trajectories were convolved with a 2D Gaussian function with fwhm of 20 fs and 0.2 eV. The theoretical temporal resolution was chosen to be higher than the experimental pump–probe cross correlation (75 fs), so as to better resolve the effects of IVR and IC on the spectrum. In addition, the active space of the trajectories was found to become unstable beyond 50 fs, meaning that simulating a TRPES spectrum with the longer experimental time resolution was not achievable. As a result, one should not directly compare the experimental and theoretical TRPES spectra, due to their different time resolutions. Nevertheless, the qualitative prediction of a faster time scale in the decay of the high vs low eKE window is robust.

Finally, it should be noted that during the dynamics, we found 8 initial conditions failed to complete due to discontinuous changes in the active space orbitals and were discarded. For comparison, 11 initial conditions failed for the comparable SA-5-CASSCF­(4,5) electronic structure. That we had to discard some failed trajectories means that one should not place too much weight on the branching ratios between the shape and Feshbach states predicted by our simulations. Nevertheless, we can draw robust qualitative conclusions from our successful trajectories that (i) HBI undergoes rapid ( <30 fs) IVR in the shape state; (ii) nonadiabatic transitions from S_3_ to S_2_ occur on a similar time scale as IVR and largely preserve the shape electronic character; (iii) internal conversion between the shape and Feshbach states occurs mainly adiabatically and reversibly on the S_2_ state.

## Supplementary Material





## Notes

Exploration of the influence of diffuse functions, *xyz* coordinates, and electronic energies of all critical point geometries (PDF)
Transparent Peer Review report available (PDF)
