# Supplementary material for: UV Response of the Green Fluorescent Protein Chromophore: Insights from Ab Initio Nonadiabatic Simulations
Source: J Phys Chem Lett. 2026 Apr 21;17(17):5005–11. doi: 10.1021/acs.jpclett.6c00415 (PMC13137242; doi:10.1021/acs.jpclett.6c00415)
Supplement: Supplementary file 1 [file jz6c00415_si_001.pdf]

Supporting Information for “UV response of the green fluorescent protein chromophore: Insights from ab initio non-adiabatic simulations”

Wutthinan Thongyod,[†,‡] Tingyao Lu,[†,¶,§] Yuanming Bai,[¶,†,§] Chutintorn Punwong,[‖,⊥] and William J. Glover[*,†,¶,§]

[†]NYU-ECNU Center for Computational Chemistry at NYU Shanghai, 3663 Zhongshan Road North, Shanghai 200062, China
[‡]School of Science, and Research Center for Theoretical Simulation and Applied Research in Bioscience and Sensing, Walailak University, Nakhon Si Thammarat 80160, Thailand
Shanghai Frontiers Science Center of Artificial Intelligence and Deep Learning; NYU Shanghai, 567 West Yangsi Road, Shanghai 200127, China
[§]Department of Chemistry, New York University, New York, New York 10003, USA
[‖]Division of Physical Science, Faculty of Science, Prince of Songkla University, Hat Yai, Songkhla 90112, Thailand
[⊥]Center of Excellence for Trace Analysis and Biosensor, Prince of Songkla University, Songkhla 90112, Thailand

E-mail: william.glover@nyu.edu

Figure S1. Electronic energies at relevant critical points on the potential energy surfaces of HBI. Panel (a) shows results at the SA-4-CAS(4,5)XMS-PT2/def2-svp level of theory, while panel (b) shows results at the SA-4-CAS(4,5)XMS-PT2/def2-svpd level (including diffuse functions). All energies are relative to S_0 at the optimized ground-state structure at the respective level of theory.

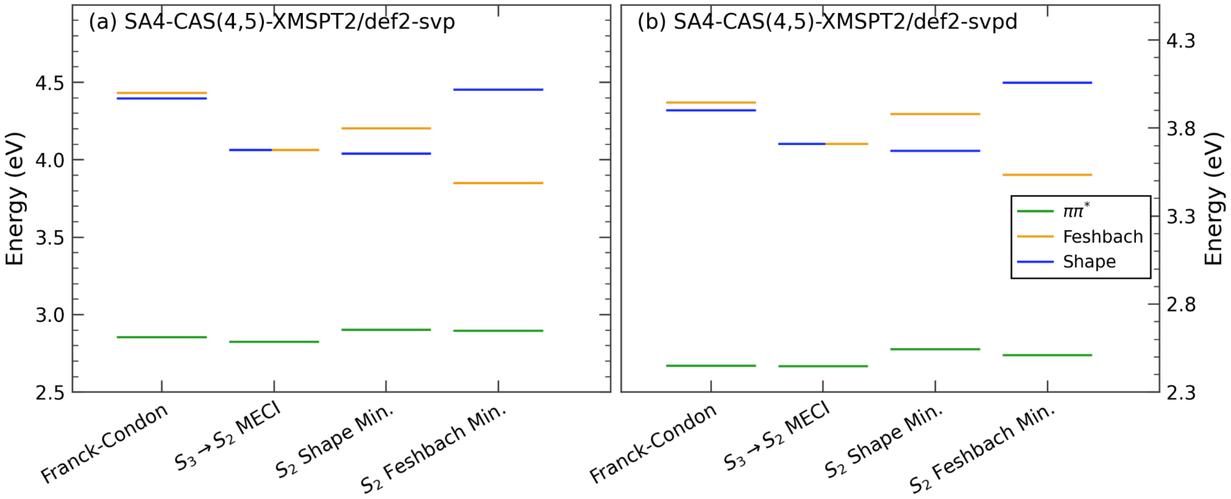

DW(4,4eV)-CASSCF(4,5)/6-31G* S_0 optimized geometry
21

N	0.0002635268	-0.0001883897	0.0116898709
N	0.0023951198	0.7804004160	-2.0627789002
H	-0.0009978562	0.0049582460	1.0042294966
H	-0.0019429436	2.0883165776	-0.4514663228
H	0.0084334184	-2.4777492577	-2.9705793406
H	0.0058262540	0.9982986333	-4.5390259333
H	0.0089798049	1.3353847789	-6.9494318838
H	0.0160182682	-2.9160157354	-7.5494433504
H	0.0128713218	-3.2432571719	-5.1376813941
C	0.0000909212	1.0860671054	-0.8364236022
C	0.0044531885	-0.6238805741	-2.1156587329
C	0.0031445421	-1.1537957475	-0.7587350050
C	0.0073269597	-1.4307204750	-3.2288962403
C	0.0090783159	-1.1542876441	-4.6167114336
C	0.0080513556	0.1483241463	-5.1956939175
C	0.0098352511	0.3395825290	-6.5383744655
C	0.0128434989	-0.7548325359	-7.4935923436
C	0.0138340788	-2.0690750772	-6.8840423913
C	0.0120237785	-2.2399391896	-5.5373616759
O	0.0041401332	-2.2775822157	-0.3145819686
O	0.0143410623	-0.5780184188	-8.7114304656

State	Energy
S_0	-641.4238165
S_1	-641.2769224
S_2	-641.2188641
S_3	-641.2157511
D_0	-641.3443286
D_1	-641.2563719

DW(4,4eV)-CASSCF(4,5)/6-31G* FC displaced geometry

21

N	0.00026243	0.00294524	0.00959166
N	0.00241766	0.77201423	-2.06995623
H	-0.00099794	0.00507045	1.00415368
H	-0.00194314	2.08826439	-0.45144252
H	0.00843344	-2.47742311	-2.97090554
H	0.00582499	0.99886315	-4.53886762
H	0.00897897	1.33576304	-6.94898289
H	0.01601916	-2.91609423	-7.54975826
H	0.01287125	-3.24308384	-5.13768687
C	0.00007366	1.08757903	-0.82628892
C	0.00442950	-0.61674223	-2.10672962
C	0.00314216	-1.14739356	-0.76339199
C	0.00733850	-1.43351919	-3.23372348
C	0.00908503	-1.15694355	-4.61889706
C	0.00808135	0.14604491	-5.21260841
C	0.00980123	0.34026501	-6.51783479
C	0.01285926	-0.77628142	-7.48102893
C	0.01380528	-2.05262678	-6.88184285
C	0.01202355	-2.23991919	-5.53693137
O	0.00414551	-2.28297826	-0.31288181
O	0.01435817	-0.57181407	-8.72997618

**State**	**Energy**
S_0	-641.4178935
S_1	-641.2740160
S_2	-641.2197532
S_3	-641.2072997
D_0	-641.3368581
D_1	-641.2541373

DW(4,4eV)-CASSCF(4,5)/6-31G* S_3/S_2 MECI geometry
21

N	0.0001770075	0.0450252510	0.0246556842
N	0.0024428269	0.7353118247	-2.0798560952
H	-0.0010917776	0.0704110030	1.0172264834
H	-0.0020013328	2.1105684266	-0.4948958858
H	0.0085692637	-2.5392322155	-2.9708544377
H	0.0058468634	0.9838546596	-4.4910064573
H	0.0089091490	1.3497352604	-6.9254169544
H	0.0161147289	-2.8896661544	-7.6165952807
H	0.0129492858	-3.2971804897	-5.1778732194
C	0.0000143419	1.0942343786	-0.8331761979
C	0.0044395499	-0.6174261032	-2.1041201066
C	0.0030392224	-1.1241630101	-0.7283691393
C	0.0074835407	-1.5000313838	-3.2423864090
C	0.0091744949	-1.1880874355	-4.6165370442
C	0.0080411647	0.1504519409	-5.1634132009
C	0.0098038658	0.3527255119	-6.5186527831
C	0.0128334193	-0.7372658709	-7.4831971360
C	0.0139013392	-2.0692729987	-6.9189929234
C	0.0121199225	-2.2928895392	-5.5665059812
O	0.0040572377	-2.2458581641	-0.2802330055
O	0.0141858861	-0.5292548918	-8.7057899100

**State**	**Energy**
S_0	-641.4145058
S_1	-641.2834985
S_2	-641.2261900
S_3	-641.2261899
D_0	-641.3435690
D_1	-641.2636186

DW(4,4eV)-CASSCF(4,5)/6-31G* S_2 Shape min. geometry
21

N	0.0002120282	0.0262228499	0.0233285098
N	0.0024208923	0.7695052639	-2.0676826743
H	-0.0010757544	0.0418678098	1.0160823408
H	-0.0019234522	2.0997583742	-0.4666804532
H	0.0085066923	-2.5079790361	-2.9889926049
H	0.0057315621	1.0008633118	-4.4836489453
H	0.0089509852	1.3649041601	-6.9552429946
H	0.0161388894	-2.9130408639	-7.6298859937
H	0.0129799734	-3.3103891205	-5.1341070523
C	0.0000724174	1.0906310288	-0.8294304533
C	0.0044288139	-0.6021161052	-2.1170616293
C	0.0030969397	-1.1316464879	-0.7460725970
C	0.0074318840	-1.4682359096	-3.2635472633
C	0.0090800858	-1.1708430224	-4.6125108751
C	0.0079572358	0.1727267573	-5.1629158273
C	0.0097509334	0.3805760080	-6.5244591767
C	0.0128076950	-0.7603832027	-7.4733798633
C	0.0139361929	-2.1010690441	-6.9245387079
C	0.0121567795	-2.3187415547	-5.5501306860
O	0.0040997287	-2.2533838120	-0.3162564903
O	0.0142494776	-0.5472374050	-8.6688565627

State	Energy
S_0	-641.3997090
S_1	-641.2677494
S_2	-641.2314924
S_3	-641.2058403
D_0	-641.3326011
D_1	-641.2457740

DW(4,4eV)-CASSCF(4,5)/6-31G* S_2 Transition State geometry

21

N	0.0002593366	0.0223474725	0.0296707410
N	0.0024240036	0.7498307836	-2.0647705039
H	-0.0011138700	0.0397414242	1.0223412413
H	-0.0019123217	2.0937003708	-0.4670287236
H	0.0085235378	-2.5204166982	-2.9897946104
H	0.0057215471	1.0244647947	-4.4984299997
H	0.0090098141	1.3499152453	-6.9787071260
H	0.0161277346	-2.9244391728	-7.6016178639
H	0.0129000058	-3.2694655099	-5.1335965091
C	0.0000646904	1.0822548279	-0.8222562319
C	0.0044409418	-0.6137807639	-2.1094061942
C	0.0031134282	-1.1382911146	-0.7372646462
C	0.0074279675	-1.4786207457	-3.2538480807
C	0.0090544128	-1.1541380691	-4.6189975488
C	0.0079505982	0.1862714827	-5.1616652238
C	0.0097908498	0.3629050712	-6.5490043917
C	0.0127769414	-0.7350731008	-7.4817399959
C	0.0139049846	-2.1014951021	-6.9105209270
C	0.0121035745	-2.2766197144	-5.5539116700
O	0.0040907879	-2.2617410209	-0.3025950537
O	0.0143510350	-0.5753592605	-8.6928391816

State	Energy
S_0	-641.4129683
S_1	-641.2804401
S_2	-641.2298064
S_3	-641.2185713
D_0	-641.3426324
D_1	-641.2612424

DW(4,4eV)-CASSCF(4,5)/6-31G* S_2 Feshbach min geometry

21

N	0.0002067768	0.0221385828	0.0358529979
N	0.0024753502	0.7250431070	-2.0658015460
H	-0.0010764287	0.0387430504	1.0285967023
H	-0.0019262348	2.0919604933	-0.4669983464
H	0.0085430561	-2.5356217027	-2.9898242242
H	0.0057470358	1.0359165089	-4.5252157080
H	0.0090320213	1.3390928237	-6.9881474916
H	0.0160578325	-2.9161688164	-7.5655841441
H	0.0128631366	-3.2431848363	-5.1423854322
C	0.0000668203	1.0781313216	-0.8115718528
C	0.0044296742	-0.6215612987	-2.1010092293
C	0.0030821040	-1.1397517212	-0.7259820462
C	0.0074294562	-1.4925254339	-3.2429911838
C	0.0090462537	-1.1414363268	-4.6204461103
C	0.0079488262	0.1863309535	-5.1741354627
C	0.0098563705	0.3476320314	-6.5650069192
C	0.0128080141	-0.7230986261	-7.4926107186
C	0.0138579860	-2.0786638580	-6.8900137422
C	0.0120477509	-2.2462267238	-5.5548441132
O	0.0040933155	-2.2668684013	-0.2888476372
O	0.0144208825	-0.5978911277	-8.7290237921

**State**	**Energy**
S_0	-641.4106004
S_1	-641.2797422
S_2	-641.2304741
S_3	-641.2122448
D_0	-641.3375359
D_1	-641.2612103

SA4-CAS(4,5)XMS-PT2/cc-pVTZ S_0 optimized geometry

21

N	0.000255059	0.020778884	-0.003379671
N	0.002493658	0.768533041	-2.116913871
H	-0.001205726	0.030968512	1.000158329
H	-0.001929216	2.115156463	-0.481501557
H	0.008418180	-2.510469979	-2.968216884
H	0.005821044	0.980044456	-4.476946310
H	0.009046352	1.353491238	-6.918102404
H	0.016061308	-2.906236100	-7.575705380
H	0.012830005	-3.268852066	-5.140588772
C	0.000069151	1.101454307	-0.850173067
C	0.004472396	-0.625293676	-2.129626701
C	0.003127022	-1.156540874	-0.770631555
C	0.007355178	-1.457648424	-3.241339481
C	0.009062236	-1.175161033	-4.609640963
C	0.008052161	0.145039677	-5.163142356
C	0.009839193	0.349431500	-6.512531380
C	0.012856786	-0.738300881	-7.480436825
C	0.013828044	-2.067219764	-6.891569826
C	0.012022125	-2.260044360	-5.540656353
O	0.004043884	-2.300500986	-0.307009313
O	0.014469553	-0.544637606	-8.710644591

**State**	**Energy**
S_0	-644.0556267
S_1	-643.9614242
S_2	-643.9092063
S_3	-643.9071711

SA4-CAS(4,5)XMS-PT2/cc-pVTZ FC displaced geometry

21

N	0.00025719	0.02338701	-0.00708476
N	0.00253181	0.75517222	-2.12971264
H	-0.00120565	0.03112636	1.00013151
H	-0.00192954	2.11501589	-0.48132313
H	0.00841768	-2.51030264	-2.96807967
H	0.00582035	0.98030207	-4.47678342
H	0.00904644	1.35325716	-6.91797964
H	0.01606046	-2.90609951	-7.57542963
H	0.01283077	-3.26880203	-5.14119072
C	0.00003564	1.10617880	-0.83254675
C	0.00444628	-0.61478440	-2.12207118
C	0.00311908	-1.14705589	-0.77433778
C	0.00736527	-1.46579339	-3.24051766
C	0.00906811	-1.17538301	-4.61330726
C	0.00807321	0.14267310	-5.17478007
C	0.00981584	0.34833955	-6.49648033
C	0.01287342	-0.73272006	-7.49632921
C	0.01380709	-2.07413049	-6.87168859
C	0.01203322	-2.25242008	-5.55468421
O	0.00405165	-2.30843903	-0.30466609
O	0.01446914	-0.54552968	-8.70973842

**State**	**Energy**
S_0	-644.0506490
S_1	-643.9575939
S_2	-643.9057107
S_3	-643.8977871
D_0	-643.9513827
D_1	-643.8887258

SA4-CAS(4,5)XMS-PT2/cc-pVTZ S_3/S_2 MECI geometry

21

N	0.000187265	0.043906562	0.018911169
N	0.002450339	0.764264915	-2.111475149
H	0.001299710	0.052489580	1.022973904
H	0.002041397	2.131765440	-0.466648448
H	0.008462378	-2.517589808	-2.961674745
H	0.005683393	0.982479424	-4.444775863
H	0.009037614	1.338444459	-6.967824164
H	0.016219806	-2.912450649	-7.618574060
H	0.012858793	-3.288248414	-5.091001964
C	0.000037640	1.114188019	-0.819562168
C	0.004421913	-0.593403515	-2.129364729
C	0.003075724	-1.126880758	-0.754601773
C	0.007393641	-1.470523854	-3.247491895
C	0.009069684	-1.175574005	-4.617517397
C	0.007936486	0.154128261	-5.135937157
C	0.009814821	0.347594882	-6.531443684
C	0.012831112	-0.758072312	-7.447347762
C	0.013957010	-2.089790741	-6.915658659
C	0.012053941	-2.291359155	-5.517323175
O	0.004065580	-2.275661604	-0.317001898
O	0.014459890	-0.555129775	-8.697140518

**State**	**Energy**
S_0	-644.0476899
S_1	-643.9615398
S_2	-643.9169380
S_3	-643.9169120
D_0	-643.9510719
D_1	-643.8954467

SA4-CAS(4,5)XMS-PT2/cc-pVTZ S_2 Shape min. geometry

21

N	-0.120674653	-0.000205198	0.010657293
N	-0.141923994	0.759390135	-2.105421772
H	-0.149943404	-0.001027042	1.014189706
H	-0.306232656	2.082766182	-0.453695989
H	0.172260064	-2.498675507	-2.996537256
H	0.066841811	1.002811779	-4.431331387
H	0.158429617	1.382073463	-6.963354779
H	-0.213389856	-2.866953336	-7.619431551
H	0.164854736	-3.275410987	-5.095005250
C	-0.202080241	1.075658993	-0.823963735
C	-0.012202692	-0.605297680	-2.142309402
C	0.005788844	-1.157512860	-0.776051825
C	0.093128744	-1.450395116	-3.267999610
C	0.113669498	-1.146191112	-4.626532186
C	0.089551960	0.184802110	-5.135531992
C	0.157679048	0.393836805	-6.522514151
C	-0.018660655	-0.731573830	-7.437587917
C	-0.073886168	-2.057950180	-6.913377880
C	0.168273379	-2.281685260	-5.526450489
O	0.101130559	-2.302132908	-0.349482972
O	-0.140235530	-0.502126922	-8.667087556

**State**	**Energy**
S_0	-644.0425872
S_1	-643.9540590
S_2	-643.9184306
S_3	-643.9064319
D_0	-643.9467940
D_1	-643.8871987

SA4-CAS(4,5)XMS-PT2/cc-pVTZ S_2 Transition State geometry

21

N	0.000197383	0.023156265	0.041236134
N	0.002587677	0.701431798	-2.117082961
H	-0.001331410	0.020002369	1.052841978
H	-0.002183914	2.142994653	-0.443795524
H	0.008466306	-2.595161231	-2.935549178
H	0.005773323	0.935482644	-4.420026188
H	0.008979079	1.377029694	-6.895444142
H	0.016162130	-2.834617622	-7.685533231
H	0.013032576	-3.310392912	-5.226262061
C	0.000000000	1.111525613	-0.785322261
C	0.004425509	-0.625396971	-2.112027732
C	0.002997789	-1.144676449	-0.714754893
C	0.007420123	-1.538600725	-3.225128705
C	0.009139420	-1.221478583	-4.623674745
C	0.008045610	0.108181284	-5.131794404
C	0.009838463	0.351269418	-6.513550709
C	0.012799209	-0.693673002	-7.470776716
C	0.013878731	-2.015049129	-6.960306980
C	0.012116571	-2.274168157	-5.582341171
O	0.003955070	-2.299724242	-0.278002188
O	0.014446009	-0.452666651	-8.755880920

**State**	**Energy**
S_0	-644.0458501
S_1	-643.9597419
S_2	-643.9170920
S_3	-643.9151431
D_0	-643.9369768
D_1	-643.8925751

SA4-CAS(4,5)XMS-PT2/cc-pVTZ S_2 Feshbach min geometry
21

N	0.000223950	0.020381583	0.026560977
N	0.002607643	0.706859122	-2.134938595
H	-0.001321903	0.014017455	1.031067214
H	-0.002575289	2.130669725	-0.449938592
H	0.008694503	-2.577685771	-2.929920363
H	0.005615261	0.916276772	-4.419876381
H	0.008629756	1.361773594	-6.867799560
H	0.016251579	-2.827727225	-7.661233891
H	0.013280676	-3.299088597	-5.218340595
C	0.000017057	1.112837715	-0.790285745
C	0.004491001	-0.619305036	-2.126232648
C	0.003074729	-1.138658023	-0.732840452
C	0.007551933	-1.533796461	-3.224071634
C	0.009200583	-1.224914319	-4.623347623
C	0.007933396	0.098298581	-5.126655022
C	0.009624765	0.344695603	-6.495504466
C	0.012677928	-0.699143810	-7.457159884
C	0.013922547	-2.013845413	-6.946559141
C	0.012241482	-2.273724473	-5.571321474
O	0.004127839	-2.296367229	-0.303257649
O	0.014218259	-0.452090488	-8.726151574

State	Energy
S_0	-644.0388749
S_1	-643.9584769
S_2	-643.9236792
S_3	-643.9031771
D_0	-643.9372842
D_1	-643.8800638

SA-4-CAS(4,5)XMS-PT2/def2-svp Franck-Condon geometry

21

N	0.000427	0.044978	0.023669
N	0.004682	1.445517	-3.974728
H	-0.002153	0.065309	1.933389
H	-0.003761	4.026662	-0.883877
H	0.016019	-4.771997	-5.594782
H	0.010875	1.866131	-8.450279
H	0.016944	2.590804	-13.106344
H	0.030463	-5.503510	-14.377636
H	0.024424	-6.213488	-9.739655
C	0.000103	2.086859	-1.587295
C	0.008460	-1.189415	-4.001862
C	0.005947	-2.192207	-1.419781
C	0.013940	-2.762317	-6.115244
C	0.017183	-2.229333	-8.720658
C	0.015145	0.278456	-9.767107
C	0.018531	0.674705	-12.329301
C	0.024241	-1.385172	-14.175954
C	0.026199	-3.910898	-13.060045
C	0.022788	-4.284272	-10.493746
O	0.007764	-4.350147	-0.543437
O	0.027229	-1.015577	-16.502266

**State**	**Energy**
S_0	-642.8944881197
S_1	-642.7895627263
S_2	-642.7329143357
S_3	-642.7316658211

SA-4-CAS(4,5)XMS-PT2/def2-svp S_3-S_2 MECI geometry
21

N 0.000295 0.089270 0.068080
N 0.004560 1.456138 -3.955408
H -0.002449 0.103684 1.979118
H -0.003985 4.060859 -0.843234
H 0.016043 -4.761742 -5.588742
H 0.010554 1.893290 -8.413794
H 0.017060 2.544813 -13.235263
H 0.030920 -5.542096 -14.439237
H 0.024339 -6.247536 -9.613455
C -0.000124 2.116754 -1.524840
C 0.008330 -1.111505 -4.002104
C 0.005810 -2.130196 -1.393301
C 0.013968 -2.763965 -6.138184
C 0.017137 -2.218311 -8.733053
C 0.014870 0.307789 -9.727070
C 0.018568 0.660549 -12.383899
C 0.024286 -1.446881 -14.106109
C 0.026555 -3.972302 -13.095646
C 0.022765 -4.346800 -10.433125
O 0.007733 -4.298362 -0.572604
O 0.027376 -1.071951 -16.472642

**State**	**Energy**
S_0	-642.8810172831
S_1	-642.7907228704
S_2	-642.7451765186
S_3	-642.74513439

SA-4-CAS(4,5)XMS-PT2/def2-svp S_2 Shape min geometry
21

N 0.000363 0.053502 0.064881
N 0.004607 1.469530 -3.941662
H -0.002343 0.060070 1.975842
H -0.003835 4.029276 -0.818277
H 0.015968 -4.736645 -5.617302
H 0.010627 1.904749 -8.401398
H 0.017200 2.584931 -13.243383
H 0.030667 -5.523338 -14.451109
H 0.024242 -6.255876 -9.585211
C -0.000005 2.092253 -1.523294
C 0.008368 -1.115521 -4.013159
C 0.005854 -2.157198 -1.412327
C 0.013932 -2.735498 -6.154851
C 0.017106 -2.189704 -8.738700
C 0.014935 0.330135 -9.729358
C 0.018624 0.707470 -12.381153
C 0.024258 -1.449843 -14.097243
C 0.026388 -3.965338 -13.092204
C 0.022722 -4.363905 -10.419107
O 0.007749 -4.325139 -0.607208
O 0.027278 -1.064016 -16.447956

State	Energy
S_0	-642.8802863161
S_1	-642.7878576887
S_2	-642.7460818393
S_3	-642.7400336114

SA-4-CAS(4,5)XMS-PT2/def2-svp S_2 Feshbach min geometry
21

N 0.000363 0.053502 0.064881
N 0.004607 1.469530 -3.941662
H -0.002343 0.060070 1.975842
H -0.003835 4.029276 -0.818277
H 0.015968 -4.736645 -5.617302
H 0.010627 1.904749 -8.401398
H 0.017200 2.584931 -13.243383
H 0.030667 -5.523338 -14.451109
H 0.024242 -6.255876 -9.585211
C -0.000005 2.092253 -1.523294
C 0.008368 -1.115521 -4.013159
C 0.005854 -2.157198 -1.412327
C 0.013932 -2.735498 -6.154851
C 0.017106 -2.189704 -8.738700
C 0.014935 0.330135 -9.729358
C 0.018624 0.707470 -12.381153
C 0.024258 -1.449843 -14.097243
C 0.026388 -3.965338 -13.092204
C 0.022722 -4.363905 -10.419107
O 0.007749 -4.325139 -0.607208
O 0.027278 -1.064016 -16.447956

State	Energy
S_0	-642.8715501719
S_1	-642.7880648776
S_2	-642.7529953022
S_3	-642.7309095748

SA-4-CAS(4,5)XMS-PT2/def2-svpd Franck-Condon geometry
21

N 0.000438 0.043784 0.017383
N 0.004753 1.416513 -3.994435
H -0.002453 0.075097 1.926412
H -0.003682 4.025696 -0.916045
H 0.016009 -4.816571 -5.617774
H 0.010904 1.852058 -8.412872
H 0.017050 2.623373 -13.055184
H 0.030544 -5.471451 -14.409241
H 0.024409 -6.225777 -9.782803
C 0.000110 2.085912 -1.603243
C 0.008476 -1.222244 -4.008464
C 0.005839 -2.199604 -1.416064
C 0.013964 -2.806353 -6.125648
C 0.017195 -2.247329 -8.721650
C 0.015179 0.278397 -9.741974
C 0.018607 0.698984 -12.304611
C 0.024292 -1.348658 -14.162482
C 0.026271 -3.889332 -13.081804
C 0.022820 -4.290942 -10.516219
O 0.007506 -4.366052 -0.512008
O 0.027258 -0.952881 -16.496538

**State**	**Energy**
S_0	-643.0252605272
S_1	-642.9352657487
S_2	-642.8819658386
S_3	-642.8802754126

SA-4-CAS(4,5)XMS-PT2/def2-svpd S_3-S_2 MECI geometry
21

N 0.000307 0.088006 0.061588
N 0.004656 1.414555 -3.979389
H -0.002568 0.118814 1.971674
H -0.003930 4.056309 -0.883684
H 0.016194 -4.824341 -5.602089
H 0.010204 1.865637 -8.364565
H 0.016879 2.592463 -13.156195
H 0.031221 -5.497077 -14.476196
H 0.024412 -6.256796 -9.679694
C -0.000101 2.109995 -1.545127
C 0.008400 -1.160965 -4.007348
C 0.005782 -2.143994 -1.383304
C 0.014048 -2.823211 -6.132678
C 0.017157 -2.241885 -8.735364
C 0.014632 0.298999 -9.696886
C 0.018485 0.691800 -12.345789
C 0.024282 -1.390907 -14.108411
C 0.026768 -3.938507 -13.121372
C 0.022812 -4.344657 -10.468845
O 0.007635 -4.318363 -0.532179
O 0.027313 -0.986078 -16.476042

**State**	**Energy**
S_0	-643.0181395991
S_1	-642.9353281909
S_2	-642.8888999686
S_3	-642.8889022621

SA-4-CAS(4,5)XMS-PT2/def2-svpd S_2 Shape min geometry
21

N 0.000379 0.051873 0.056299
N 0.004689 1.436083 -3.964619
H -0.002461 0.073695 1.966315
H -0.003754 4.025429 -0.862265
H 0.016056 -4.791175 -5.636990
H 0.010266 1.877228 -8.349198
H 0.016976 2.634027 -13.167588
H 0.031068 -5.484782 -14.489563
H 0.024342 -6.273191 -9.640718
C 0.000035 2.086286 -1.549079
C 0.008420 -1.162098 -4.019386
C 0.005814 -2.171781 -1.407070
C 0.013969 -2.785869 -6.153130
C 0.017102 -2.211110 -8.740175
C 0.014703 0.324392 -9.699229
C 0.018527 0.742857 -12.339042
C 0.024296 -1.404654 -14.096386
C 0.026643 -3.939379 -13.117762
C 0.022756 -4.371987 -10.450184
O 0.007614 -4.343094 -0.569592
O 0.027268 -0.980483 -16.445545

State	Energy
S_0	-643.0170813339
S_1	-642.9317292457
S_2	-642.8903517301
S_3	-642.8827057919

SA-4-CAS(4,5)XMS-PT2/def2-svpd S_2 Feshbach min geometry
21

N 0.000301 0.091436 0.062801
N 0.004871 1.322778 -4.033913
H -0.002679 0.115355 1.973642
H -0.004083 4.085060 -0.897240
H 0.016347 -4.932371 -5.534395
H 0.010072 1.743063 -8.326093
H 0.016692 2.616597 -12.986063
H 0.031119 -5.357789 -14.535731
H 0.024548 -6.278928 -9.893180
C -0.000187 2.132658 -1.521589
C 0.008457 -1.200614 -3.999406
C 0.005692 -2.134772 -1.340746
C 0.014189 -2.937583 -6.085236
C 0.017324 -2.331310 -8.740752
C 0.014617 0.191492 -9.681408
C 0.018496 0.672525 -12.286787
C 0.024216 -1.304084 -14.116196
C 0.026630 -3.813101 -13.163681
C 0.022756 -4.319338 -10.554594
O 0.007554 -4.320258 -0.480835
O 0.027341 -0.828557 -16.530565

**State**	**Energy**
S_0	-643.0103307936
S_1	-642.9329897348
S_2	-642.8953752816
S_3	-642.8761744692
