# Supplementary material for: UV Response of the Green Fluorescent Protein Chromophore: Insights from Ab Initio Nonadiabatic Simulations
Source: J Phys Chem Lett. 2026 Apr 21;17(17):5005–11. doi: 10.1021/acs.jpclett.6c00415 (PMC13137242; doi:10.1021/acs.jpclett.6c00415)
Supplement: Supplementary file 2 [file jz6c00415_si_002.pdf]

jz-2026-004159.R1

Name: Peer Review Information for "UV response of the green fluorescent protein chromophore: Insights from ab initio non-adiabatic simulations"

First Round of Reviewer Comments

Reviewer: 1

Comments to the Author

In this manuscript, the authors tried to simulate the excited-state dynamics and time-resolved photoelectron spectroscopy of the green fluorescent protein chromophore. The ab initio multiple spawning method coupled with on-the-fly multiconfigurational electronic structure using the dynamically weighted complete active space self-consistent field method was used. It was found that the conversion between the shape and Feshbach states occurs primarily adiabatically on the S2 state. The motivation of studying the UV response of the green fluorescent protein chromophore is sound, and the story looks reasonable. Thereby, I would like to recommend the manuscript to be published in J. Phys. Chem. Lett. after the following points are considered.

(1) On page 2, the authors stated that “Given the complications of overlapping UV absorption bands from the protein environment, a bottom-up approach to understanding GFP’s photo-oxidation has been popular, focusing on the photoresponse of the isolated p-hydroxybenzilidene-2,3-dimenthylimidazolinone (HBDI) chromophore anion and related biomimetic molecules.” This sounds reasonable. However, the protein environment may still play important roles when studying the mechanism of UV response of green fluorescent protein as the potential energy surfaces are closely related to the protein environment. On page 3, the authors also mentioned that “in the gas phase, the initial photo-oxidation occurs via autodetachment, while in the protein environment, it occurs via a charge-transfer reaction, possibly forming a hydrated electron”. This actually means the protein environment does play important roles. As an isolated HBI is studied in this manuscript, some discussions are needed.

(2) It would be better if the experimental results of time-resolved photoelectron spectroscopy can be directly compared with the simulated results in Fig. 3 if possible.

(3) On page 5, the authors stated that “We identify a minimum on the S2 surface with shape character (S2 Shape Min.), leading to a rather large reorganization energy of 0.66 eV from the FC region and an even larger change of the shape state energy relative to the vertical D0 state (1.53 eV to 0.75 eV)”. How is the reorganization energy calculated here?

(4) Among the chosen 20 initial conditions, 7 initial conditions failed to complete due to discontinuous changes in the active space orbitals and were discarded. Does this influence the results and mechanisms proposed in this manuscript?

Reviewer: 2

Comments to the Author

1. What is the major advance reported in the paper?

This work offers a theoretical investigation of the photodynamics of a reduced model of the anionic GFP chromophore (HBI). It shows that two diverging interpretations of earlier TRPES experiments can be reconciled by an ultrafast (and diabatic) conversion from the photoexcited S3 state to S2, followed by an adiabatic evolution between shape- and Feshbach-state characters in S2.

2. What is the immediate significance of this advance?

This work clarifies the mechanisms of deactivation of GFP chromophores following UV excitation, providing strategies to improve the photostability of these chromophores.

3. Technical suggestions

This work reports solid simulations of excited-state dynamics and TRPES signals. The text is well-written, and the authors did a great job at highlighting the difference between adiabatic states and their underlying characters, key to a reconciliation of the TRPES interpretation. I only have a few minor comments for the authors:

(a) The calculations focus on the four lowest electronic states. Given that the dynamics is initiated in the highest electronic state considered (S3), have the authors checked whether higher singlet states (S4, S5, etc) are close in energy and could interact with S3 in the photodynamics?

(b) The authors comment on the energy shift resulting from the neglect of dynamic correlation with CASSCF-based methods. I was wondering whether they would observe a similar shift if they were to include diffuse functions in their basis set. I realize the challenge of including such functions to study the excited states of an anion, but given the rather different electronic character of the excited states considered, I was wondering whether the authors could provide reassurance that a larger basis set would solely result in a rigid shift of the electronic energies for the states considered.

(c) It might be interesting to provide the details of the pump pulse the authors have tried to mimic with their selection of initial conditions (possibly for future experiments on this system).

Author's Response to Peer Review Comments:

We thank both reviewers for their helpful comments, which we have addressed in their entirety. We believe that our manuscript is now stronger and hope that it will be found suitable for publication.

The reviewers' original comments are reproduced here in black text. Our response to each comment is in blue. Changes made to the manuscript have been highlighted with red text, both here and in a supplementary marked-up copy of the manuscript.

Reviewer(s)' Comments to Author:

Reviewer: 1

Recommendation: This paper may be publishable, but major revision is needed; I would like to be invited to review any future revision.

Comments:
In this manuscript, the authors tried to simulate the excited-state dynamics and time-resolved photoelectron spectroscopy of the green fluorescent protein chromophore. The ab initio multiple spawning method coupled with on-the-fly multiconfigurational electronic structure using the dynamically weighted complete active space self-consistent field method was used. It was found that the conversion between the shape and Feshbach states occurs primarily adiabatically on the S2 state. The motivation of studying the UV response of the green fluorescent protein chromophore is sound, and the story looks reasonable. Thereby, I would like to recommend the manuscript to be published in J. Phys. Chem. Lett. after the following points are considered.

We sincerely thank the reviewer for their helpful comments and appraisal of our manuscript.

(1) On page 2, the authors stated that "Given the complications of overlapping UV absorption bands from the protein environment, a bottom-up approach to understanding GFP's photo-oxidation has been popular, focusing on the photoresponse of the isolated p-hydroxybenzilidene-2,3-dimenthylimidazolinone (HBDI) chromophore anion and related biomimetic molecules." This sounds reasonable. However, the protein environment may still play important roles when studying the mechanism of UV response of green fluorescent protein as the potential energy surfaces are closely related to the protein environment. On page 3, the authors also mentioned that "in the gas phase, the initial photo-oxidation occurs via autodetachment, while in the protein environment, it occurs via a charge-transfer reaction, possibly forming a hydrated electron". This actually means the protein environment does play important roles. As an isolated HBI is studied in this manuscript, some discussions are needed.

We agree that the protein environment could play an important role in GFP's photo-oxidation; however, our focus in this manuscript is on the <50-fs dynamics of the chromophore that precedes photo-oxidation, and for reasons below, we expect the protein will not completely disrupt the internal conversion mechanism we have uncovered, but may alter the extent of the internal conversion. Following the reviewer's advice, we have added the following paragraphs to the end of the manuscript text to better clarify how the gas-phase results might relate to the protein:

"Following a bottom-up approach, we chose to focus on the UV-initiated excited-state dynamics of the gas-phase GFP chromophore that precedes photo-oxidation. It is natural to anticipate how our findings might relate to the dynamics of the chromophore in the protein. Since we find that IVR and IC occur within 50 fs, significant protein reorganization will not take place on this timescale. Furthermore, our critical point calculations reveal that the chromophore in its S_2 and

S_3 states stays largely planar, so steric effects of the protein are also likely not important. We thus expect that studies of the UV-initiated intrinsic early-time dynamics of the isolated chromophore can be useful also for understanding the photophysics of the protein.

Nevertheless, the protein electrostatic and hydrogen-bonding environment could alter the vertical and adiabatic energies of the states that correlate with the gas-phase shape- and Feshbach states, which could modify the extent of internal conversion revealed by gas-phase studies. Bochenkova et al found that the shape state's vertical excitation shifted from ~325 nm (3.8 eV) in the gas phase to ~280 nm (4.4 eV) in the protein.[15] The gas-to-protein shift of the Feshbach state was not reported, but given the greater charge-transfer character of the shape state, the shift for the Feshbach state is expected to be smaller in magnitude. This will lead to an increase in the shape state's energy relative to the Feshbach state which, based on our picture, would lead to more complete IC from shape to Feshbach. We are thus currently testing this hypothesis by conducting excited-state dynamics simulations of the chromophore in its protein environment and we will report those results in a future publication."

(2) It would be better if the experimental results of time-resolved photoelectron spectroscopy can be directly compared with the simulated results in Fig. 3 if possible.

We have added the t = 25fs experimental TRPES spectrum from the West et al study to what is now figure 2(a), where good agreement is seen with our simulated spectrum:
"Figure 2: TRPES of the gas-phase HBI anion with pump and probe photon energies of 4.1 eV and 1.55 eV respectively. (a) Experimental data, digitized from Fig. 2(a) of Ref. 16. To aid the comparison to the theoretical spectra, the electron kinetic energy range is restricted to 2.0 to 3.5 eV. (b) Theoretical spectra from the DW(4,4eV)-CASSCF(4,5)/6-31G* simulations described in this paper. (c) Breakdown of the TRPES in terms of state character."

We are unable to make a *fully direct* comparison of the simulated and experimental TRPES since we used a 20-fs time resolution in convolving the simulated TRPES with a temporal Gaussian function, while the experimental pump-probe cross correlation time was 75 fs. Our simulations terminated at 50fs, which is comparable to the experimental time resolution. We would need longer simulations in order to convolve the simulated TRPES with a Gaussian function of FWHM of 75 fs and not have the spectral dynamics truncated. This was not possible because trajectories terminated beyond 50 fs due to active space instabilities. Nevertheless, our simulated spectral dynamics are fully consistent with the observations of the West et al study, which fit an instrument-response limited decay of the TRPES signal between 2.5 at 3.0 eV. Our simulations show this region of the TRPES decays on a ~20-fs timescale mainly due to IVR, which is a new interpretation of the TRPES.

We have added an expanded discussion of the TRPES temporal resolution in the Computational Methods section:
"Finally, the raw TRPES signals from the trajectories were convolved with a 2D Gaussian function with FWHM of 20 fs and 0.2 eV. The theoretical temporal resolution was chosen to be

higher than the experimental pump-probe cross correlation (75 fs), so as to better resolve the effects of IVR and IC on the spectrum. In addition, the active space of the trajectories was found to become unstable beyond 50 fs, meaning that simulating a TRPES spectrum with the longer experimental time resolution was not achievable. As a result, one should not directly compare the experimental and theoretical TRPES spectra, due to their different time resolutions. Nevertheless, the qualitative prediction of a faster timescale in the decay of the high vs low eKE window is robust."

(3) On page 5, the authors stated that "We identify a minimum on the S2 surface with shape character (S2 Shape Min.), leading to a rather large reorganization energy of 0.66 eV from the FC region and an even larger change of the shape state energy relative to the vertical D0 state (1.53 eV to 0.75 eV)". How is the reorganization energy calculated here?

The reorganization energy is the difference between Franck-Condon vertical shape energy and shape S_2 minimum energy. In the manuscript, we have added the following:
"We identify a minimum on the S_2 surface with shape character (S_2 Shape Min.), leading to a rather large reorganization energy of 0.66 eV (the difference between FC vertical shape energy and shape S_2 minimum energy) from the FC region and an even larger change of the shape state energy relative to the vertical D_0 state (1.53 eV to 0.75 eV)"

(4) Among the chosen 20 initial conditions, 7 initial conditions failed to complete due to discontinuous changes in the active space orbitals and were discarded. Does this influence the results and mechanisms proposed in this manuscript?

Failed trajectories are a very common phenomenon in excited-state dynamics studies, especially with CASSCF electronic structure. The conclusions of the paper are not affected by excluding some trajectories: had the trajectories not failed, there would still be excited-state population that is split between shape and Feschbach character on the S_2 state. We cannot however make quantitative claims about the branching ratios of the two states, nor can we rule out the possibility of other simultaneous photodeactivation pathways. Neither of these claims were made in the original manuscript. We have expanded the discussion about failed trajectories in the Computational Methods section of the manuscript (note: we made a typo- it should have been 8 failed initial conditions):

"That we had to discard some failed trajectories means that one should not place too much weight on the branching ratios between shape and Feshbach states predicted by our simulations. Nevertheless, we can draw robust qualitative conclusions from our successful trajectories that (i) HBI undergoes rapid (< 30 fs) IVR in the shape state; (ii) non-adiabatic transitions from S_3 to S_2 occur on a similar timescale as IVR and largely preserve the shape electronic character; (iii) internal conversion between the shape and Feshbach states occurs mainly adiabatically and reversibly on the S_2 state."

Additional Questions:
Urgency: High

Significance: High

Novelty: Top 10%

Scholarly Presentation: High

Is the paper likely to interest a substantial number of physical chemists, not just specialists working in the authors' area of research?: Yes

Reviewer: 2

Recommendation: This paper is publishable subject to minor revisions noted. Further review is not needed.

Comments:
1. What is the major advance reported in the paper?

This work offers a theoretical investigation of the photodynamics of a reduced model of the anionic GFP chromophore (HBI). It shows that two diverging interpretations of earlier TRPES experiments can be reconciled by an ultrafast (and diabatic) conversion from the photoexcited S3 state to S2, followed by an adiabatic evolution between shape- and Feshbach-state characters in S2.

2. What is the immediate significance of this advance?

This work clarifies the mechanisms of deactivation of GFP chromophores following UV excitation, providing strategies to improve the photostability of these chromophores.

3. Technical suggestions

This work reports solid simulations of excited-state dynamics and TRPES signals. The text is well-written, and the authors did a great job at highlighting the difference between adiabatic states and their underlying characters, key to a reconciliation of the TRPES interpretation. I only have a few minor comments for the authors:

We sincerely thank the reviewer for positive comments.

(a) The calculations focus on the four lowest electronic states. Given that the dynamics is initiated in the highest electronic state considered (S3), have the authors checked whether higher singlet states (S4, S5, etc) are close in energy and could interact with S3 in the photodynamics?

States S_4-S_{12} were included in our electronic structure; however, no non-adiabatic transitions to these states were observed in any of our trajectories. We have clarified the number of states included in the Computational Methods section:

"The width of the cubic spline was 4 eV. In total, 12 excited state were included in the simulation."

and pointed out the absence of upward non-adiabatic transitions in the paragraph before Figure 3:

"Although included in our electronic structure calculations, no transitions to S_4 or higher were noted. We do observe a small population on S_1 by 50 fs, ..."

(b) The authors comment on the energy shift resulting from the neglect of dynamic correlation with CASSCF-based methods. I was wondering whether they would observe a similar shift if they were to include diffuse functions in their basis set. I realize the challenge of including such functions to study the excited states of an anion, but given the rather different electronic character of the excited states considered, I was wondering whether the authors could provide reassurance that a larger basis set would solely result in a rigid shift of the electronic energies for the states considered.

In the SI, we now report XMS-PT2 critical points with the def2-svp and def2-spvd basis sets, the latter of which include diffuse functions. Including diffuse functions is seen to shift down the excitation energies by only ~0.33 eV and these shifts are very nearly uniform with geometry and shape/Feshbach state. Thus, the omission of diffuse basis functions in our AIMS simulations is indeed expected to result in a rigid shift of the electronic energies for the states under consideration.

In addition to the new figure S1, we have expanded the discussion about the energy shift in the Computational Methods section:

"We confirmed that although the ground-to-excited state energies of HBI are significantly overestimated at the CASSCF level, due largely to a neglect of dynamic electron correlation, the relative excited-state energies at key critical points are in good agreement with high-level multireference perturbation theory calculations at the SA-4-CAS(4,5)XMS-PT2/cc-pVTZ level (and SA4-CAS(3,5)-XMS-PT2 for the ionized neutral states) using the BAGEL quantum chemistry package[26] (see fig. 4). We also verified that augmentation of the basis set with diffuse

functions, while lowering the excitation energies of all states by around 0.33 eV, does so uniformly across all relevant critical point geometries (see fig. S1). Taken together, this suggests a good degree of parallelity between excited-state potential energy surfaces computed with DW-CASSCF without diffuse functions and high-level XMS-PT2 with diffuse functions. Thus, improvement of the electronic structure method is not expected to qualitatively change the conclusions of our study."

(c) It might be interesting to provide the details of the pump pulse the authors have tried to mimic with their selection of initial conditions (possibly for future experiments on this system).

This was mentioned in the original Computational Methods section, but we realize details of the TRPES protocol were spread over a few paragraphs. We have re-written this section to hopefully make the TRPES protocol more clear:

"To simulate time-resolved photoelectron spectra, we followed the protocol of Ref. 27, while mimicking the experimental conditions of a 4.1eV pump and 1.55 eV probe.16 The initial ground-state wavepacket was expanded in a basis of 20 Gaussian trajectory basis functions (TBF), each sampled from the Wigner distribution of HBI under the harmonic approximation using frequencies calculated at the MP2/6-31G** level. The initial TBFs were further enforced to have a ground-excited vertical energy gap of 0.216±0.0005 Hartree (5.88±0.014 eV) in order to mimic the experimental pump pulse that was slightly blue of the Franck-Condon (FC) S_3 action-spectra peak,[16] taking into account that the anionic CASSCF excitation energies are overestimated relative to the neutral D_0 state. The wavepacket was then projected on to the excited states, with each TBF amplitude weighted according to its ground-excited oscillator strength, and then uncoupled, following the independent first-generation approximation.[24] Due to the pump energy window, only the S_3 state was populated among the initial TBFs. Ab Initio multiple spawning dynamics[19,24] was then propagated with adaptive timesteps of 20 a.u. by default, but steps were rejected and repeated with half the previous timestep if CI vector overlaps involving the occupied state between steps were not diagonally dominant or energy discontinuities greater than 0.006 Hartree (0.16 eV) were detected.

To generate TRPES spectra, the anionic wavepacket was projected to the neutral doublet states. The electron kinetic energy for each TBF's contribution to the TRPES was evaluated as:

$EKE = h\nu_{probe} - (E_{neut} - E_{anion} + \Delta)$, (1)

where $h\nu$ = 1.55 eV and Δ is an energetic shift of +2.0 eV applied to the neutral doublet state to bring the TRPES spectrum into alignment with experiment. This shift, also applied in Fig. 4(a), mainly corrects for the overestimation of the anionic excited-state energies at the CASSCF level (and also from the lack of diffuse functions in our calculations)."

Additional Questions:
Urgency: High

Significance: Top 10%

Novelty: High

Scholarly Presentation: Top 10%

Is the paper likely to interest a substantial number of physical chemists, not just specialists working in the authors' area of research?: Yes

Editorial Formatting Changes requested:

1. Please provide a brief, nonsentence description of the actual contents of each Supporting Information file.

This has been addressed in the manuscript and the uploaded file description:
"Exploration of the influence of diffuse functions, xyz coordinates and electronic energies of all critical point geometries."

2. Please include author names, article titles, journal name, and publication year for the following incomplete journal reference: 11.

This has been fixed.
In addition to the above, we made some minor clarifications and changes to the language of the manuscript in a few places:

replacing "Vibrational Energy Relaxation (VER)" with "Intramolecular Vibrational Energy Redistribution (IVR)" throughout.

P5: "The simulated TRPES shows that the photoelectron signal between 2.5 and 3.0 eV is seen to decay on a timescale faster than the photoelectron signal between 2.0 and 2.5 eV in line with the experimental observations."

P5: "the Feshbach state is dark to one-photon ionization to D_0, as expected from its low Dyson norm, and ionization to D_1 is not observed in this eKE range."

P6: "Figure 5: Potential energy surfaces in the branching plane of the S_3/S_2 minimal energy conical intersection computed with DW(4,4eV)-CASSCF(4,5)/6-31G*."

P7: “While this is not the case in the isolated GFP anion since the Feshbach and shape states have similar threshold energies and rapidly interconvert, “
